# The Effect of Blood Flow Restriction Exercise on Angiogenesis-Related Factors in Skeletal Muscle Among Healthy Adults: A Systematic Review and Meta-Analysis

**DOI:** 10.3389/fphys.2022.814965

**Published:** 2022-02-17

**Authors:** Shuoqi Li, Shiming Li, Lifeng Wang, Helong Quan, Wenbing Yu, Ting Li, Wei Li

**Affiliations:** ^1^School of Health Science, Universiti Sains Malaysia, Kelantan, Malaysia; ^2^Institute of Sports Human Science, Ocean University of China, Shandong, China; ^3^Exercise and Metabolism Research Center, College of Physical Education and Health Sciences, Zhejiang Normal University, Zhejiang, China

**Keywords:** blood flow restriction, angiogenesis, skeletal muscle, resistance exercise, healthy adults

## Abstract

**Background:**

Blood flow restriction (BFR) exercise may be a potential exercise program to promote angiogenesis. This review aims to compare the effects of exercise with and without BFR on angiogenesis-related factors in skeletal muscle among healthy adults.

**Methodology:**

Searches were made in Web of Science, Scopus, PubMed, and EBSCO databases from January 2001 to June 2021. Studies were screened, quality was evaluated, and data were extracted. The review protocol was registered at PROSPERO (PROSPERO registration number: CRD42021261367). Standardized mean differences (SMD) of vascular endothelial growth factor (VEGF), vascular endothelial growth factor receptor 2 (VEGFR-2), hypoxia inducible factor 1α (HIF-1α), peroxisome proliferator-activated receptorγcoactivator-1α (PGC-1α) and endothelial nitric oxide synthase (eNOS) were analyzed using Revman 5.4 software with a 95% confidence interval (95% CI).

**Results:**

Ten studies fulfilled the inclusion criteria with a total of 75 participants for BFR group and 77 for CON group. BFR exercise elicits greater expression of VEGF (heterogeneity test, *P* = 0.09, I^2^ = 44%; SMD, 0.93 [0.38, 1.48], *P* < 0.05), VEGFR-2 (heterogeneity test, *P* = 0.81, I^2^ = 0%; SMD, 0.64 [0.08, 1.21], *P* < 0.05), HIF-1α (heterogeneity test, *P* = 0.67, I^2^ = 0%; SMD, 0.43 [0.03, 0.82], *P* < 0.05), PGC-1α (heterogeneity test, *P* = 0.02, I^2^ = 54%; SMD, 0.74 [0.21, 1.28], *P* < 0.05) and eNOS (heterogeneity test, *P* = 0.88, I^2^ = 0%; SMD, 0.60 [0.04, 1.17], *P* < 0.05) mRNA than non-BFR exercise. In the sub-group analysis, resistance exercise with BFR elicits greater expression of VEGF (heterogeneity test, *P* = 0.36, I^2^ = 6%; SMD, 1.66 [0.97, 2.35], *P* < 0.05) and HIF-1α (heterogeneity test, *P* = 0.56, I^2^ = 0%; SMD, 0.51 [0.01, 1.02], *P* < 0.05) mRNA than aerobic exercise with BFR.

**Conclusion:**

Exercise with BFR elicited more angiogenesis-related factors mRNA expression than exercise without BFR, but not VEGF and PGC-1α protein expression. Therefore, BFR training may be a potential training program to improve vascular function.

**Systematic Review Registration:**

[https://www.crd.york.ac.uk/prospero/], identifier [CRD42021261367].

## Introduction

Blood flow restriction (BFR) can limit the blood flow and increase the mechanical pressure to the working muscle during exercise, resulting in local hypoxia/ischemia. Low-load training with BFR is more conducive to increasing muscle strength (Lixandrão et al., 2018) and endurance (Kacin and Strazar, 2011) than that without BFR, which may be attributed to improved oxygen delivery and extraction of working skeletal muscles during BFR training. The combination of these mechanisms increases the peripheral vascular system adaptability (Kacin and Strazar, 2011). A recent meta-analysis demonstrated that resistance training impacts more positively on arterial compliance regulation as the capacity to restrict blood flow increases (Liu et al., 2021). Compared to traditional resistance training, this positive effect on vascular function was significantly higher when the training time does not exceed four weeks (Liu et al., 2021). Nevertheless, the relevant mechanism of BFR-training regulating the peripheral vascular system (PVS) remains unclear.

The neogenesis of peripheral capillaries has been suggested as one of the underlying mechanisms through which BFR-training regulates the PVS. A previous study that indirectly assessed the skeletal muscle microvasculature using the capillary filtration technique showed that BFR exercise enhanced capillary growth (Hunt et al., 2013). In the study of Mueller et al. (2014), 21 young men were divided into two groups for 8 weeks of resistance training intervention. Their results showed that the synergism of whole-body vibration and blood flow restriction could further improve the capillary-to-fiber ratio, which were not observed by resistance training alone. Furthermore, the expression of some angiogenic genes was reported to be significantly enhanced following low-load resistance exercise with BFR (Larkin et al., 2012). The increase of angiogenesis gene expression is closely related to angiogenesis after exercise (Olfert et al., 2016). Therefore, further analysis is required to determine the physiological or molecular mediators of this response.

One of the principal growth factors in the complex pathways involved in angiogenesis is the vascular endothelial growth factor (VEGF) (Olfert et al., 2010). During low-load resistance exercise with BFR, the resulting decrease in muscle oxygen levels may stabilize hypoxia-inducible factor 1α (HIF-1α) for targeted activation of VEGF transcription (Barjaste et al., 2021). Furthermore, VEGF efflux from the skeletal muscle was promoted through the activity of endothelial nitric oxide synthase (eNOS) after the generation of nitric oxide due to shear stress (Gielen et al., 2011). These events improved the availability of VEGF at EC-receptor sites for vascular endothelial growth factor receptor 2 (VEGFR-2) activation (Shen et al., 1998) and facilitated the angiogenic effect of VEGF (Milkiewicz et al., 2005), whereas the expression of skeletal muscle VEGF was mainly mediated by peroxisome proliferator-activated receptor-gamma co-activator alpha (PGC-1α) (Leick et al., 2009). Conclusively, VEGF secretion may be regulated by BFR in various ways to promote angiogenesis.

There is data paucity regarding the effect of BFR exercise on various angiogenesis-related factors in skeletal muscle. Research findings have demonstrated that BFR exercise could facilitate the expression of VEGF by enhancing HIF-1α (Larkin et al., 2012; Ferguson et al., 2018), whereas other researchers have contradicting views (Taylor et al., 2016; Preobrazenski et al., 2020). This review was conducted to compare the effects of BFR exercise and non-BFR on angiogenesis-related factors, and to explore the effects of various exercise programs on VEGF and HIF. The findings will improve the current body of knowledge on the role of BFR exercise in angiogenesis.

## Methodology

### Protocol and Registration

The review protocol was registered on June 18, 2021, with the International Platform of Registered Systematic Review and Meta-Analysis Protocols (PROSPERO registration number: CRD42021261367).

### Data Sources and Study Selection

Because the test methods and experimental instruments in the early research are very different from those now, the research in recent twenty years is searched. The systematic search was conducted using four databases: Scopus, PubMed, Web of Science and EBSCO. Studies published between January 2001 and June 2021 were considered while the last retrieval date was June 18, 2021. The search terms used were “blood flow restriction,” “kaatsu,” “blood flow restricted,” “HIF,” “VEGF,” “NOS,” “PGC,” “training,” and “exercise.” Both the search strategy for each database and the corresponding results are presented in Appendix A. To minimize bias during the literature search, the titles and abstracts retrieved from the databases were screened by two independent investigators.

**FIGURE 1:**
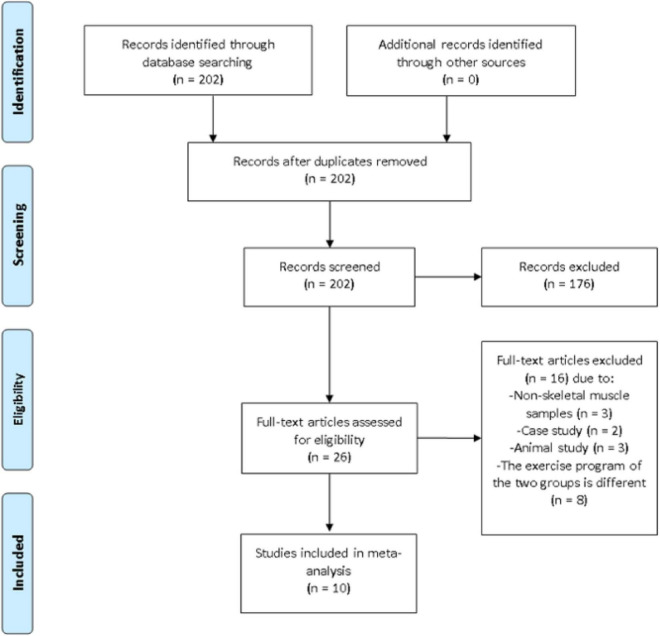
Preferred reporting items for systematic reviews and meta-analysis (PRISMA) flow diagram of search results.

Additional relevant information such as the first author’s name, the year of publication, sample size, exercise program, age, BFR method and main findings were documented. Outcome variables included VEGF, VEGFR-2, PGC-1α, HIF-1α, and eNOS. When necessary, the corresponding authors were contacted via e-mail to clarify any unclear information. A third investigator was assigned to provide an opinion if the two principal investigators disagree on any information from the articles. On that note, the disagreement was resolved after discussing and reaching a consensus. The article selection process is summarized in Figure 1.

### Inclusion and Exclusion Criteria

The inclusion criteria entailed full-text articles written in English, cross-sectional study design, participants were healthy individuals between 18 and 40 years old, the experimental group was exercise combined with BFR while the control (CON) group was exercise without BFR. Studies were also eligible for inclusion if the sampling method entailed vastus lateralis muscle biopsy taken either before exercise or two to four hours after exercise. Other literature such as abstracts, conference proceedings, posters, or presentations were excluded.

### Quality Assessment

A pre-designed quality assessment tool, the NIH quality assessment tool for observational cohort and cross-sectional studies, was used to assess all the included studies. Study Quality Assessment Tools -- NHLBI^1^, accessed 15 June 2020) has a total of 14 questions. The quality of each study was classified as “poor,” “fair,” or “good.”

### Risk of Bias Assessment

A funnel plot was used to analyze the publication bias of the study. Sensitivity analysis was performed if more than five studies include the same indicator. Specifically, the sensitivity analysis was conducted by excluding each study sequentially to determine the stability of the meta-analysis results.

### Data Analysis

The meta-analysis was executed by entering all the relevant outcome variables in the Review Manager (Version 5.4, Copenhagen: The Nordic Cochrane Center, The Cochrane Collaboration, 2014). All the studies included in the analysis had continuous outcome variables while the test units and methods were different. Therefore, standardized mean difference (SMD) was chosen as the effect scale index. Heterogeneity between studies was tested using the I (Kacin and Strazar, 2011) statistic. Heterogeneity was considered absent between studies when the I (Kacin and Strazar, 2011) is less than 40%. Therefore, a fixed-effect model can be used for analysis. Conversely, heterogeneity is present between studies if I (Kacin and Strazar, 2011) is equal to or greater than 40% and a random effect model must be used for analysis. Sub-group analysis was further performed to determine the heterogeneity. The level of statistical significance was adjusted to *P* < 0.05. According to the Cohen’s guideline (Cohen, 1992), the effect size was explained as: 0.2 is a small effect, 0.5 is a medium effect and 0.8 is a large effect.

## Results

### Eligibility of Studies

A total of 10 cross-sectional studies evaluating the effects of BFR training on angiogenesis-related factors in skeletal muscle among healthy adults were included in this review. The basic information in the included studies is shown in Table 1. Ethical approvals were obtained from the various institutions in which the studies were conducted. The level of agreement between the two principal investigators was high with a Cohen kappa coefficient of 0.884. A total of 74 men and 3 women participated in the studies, corresponding to the overall sample size in the BFR and CON groups. Seven studies focused on PGC-1α and VEGF. HIF-1α were reported in six articles while only three studies evaluated VEGFR-2 and eNOS. Six of the articles employed resistance exercises, whereas aerobic exercises were used in the remaining four studies. Overall, the BFR pressure ranged from 50 to 220 mmHg.

**TABLE 1:** Overview of the included studies.

Author	Sample size	Age(y)	Exercise program	Blood flow restriction	Outcomes	Test timing (h)	Test method
AE							
Barjaste et al., 2021	5M	33.4 ± 1.0	5 × 2 min aerobic walking with 40%VO_2*max*_; 1-min rest between each repetition.	7-cm wide Inflatable cuffs; 200 mmHg	PGC-1α; VEGF; HIF-1α	0&2	WB
Christiansen et al., 2018	6M	26.0 ± 5.0	9 × 2 min aerobic running with 12km/h; 1-min rest between each repetition; 34 min.	Inflatable cuff; 175 mmHg	PGC-1α	0&3	PCR
Preobrazenski et al., 2020	12M	22.4 ± 3.0	Cycled upright with legs below the heart;77%HR_*max*_; 30 min.	Cycled supine with legs above the heart	PGC-1α; VEGFA; HIF-1α	0&3	PCR
Taylor et al., 2016	8M	32.0 ± 7.0	Four 30 s “all-out” sprints; 4.5 min recovery.	Inflatable cuff; 130 mmHg	PGC-1α; VEGF; VEGFR-2; HIF-1α; eNOS	0&3	PCR
RE							
Ameln et al., 2005	9M	22.0 ± 2.0	Knee extension; 45 min; 26% of peak load.	Pressure chamber; 50 mmHg	VEGF; HIF-1α	0&2	PCR
Drummond et al., 2008	6M	32.0 ± 2.0	Knee extension; 20%RM; 4 sets (30, 15, 15, 15); 30 s recovery.	Inflatable cuff; 200 mmHg	HIF-1α	0&3	PCR
Ferguson et al., 2018	6M	26.0 ± 2.0	Bilateral knee extension; 20%RM; 4sets (30:15:15: continued to fatigue); 30 s recovery.	13 cm wide Inflatable cuffs; 110 mmHg	PGC-1α; VEGF; VEGFR-2; HIF-1α	0,2&4	WB; PCR
Larkin et al., 2012	3M/3F	22.0 ± 1.0	knee extension;40%RM; 10sets × 12repetitions; 60s recovery.	Inflatable cuff; 220 mmHg	PGC-1α; VEGF; VEGFR-2; HIF-1α; eNOS	0&4	WB; PCR
Norrbom et al., 2004	9M	23.0 ± 2.0	Knee extension; 45 min; 26% of peak load.	Pressure chamber; 50 mmHg	PGC-1α	0&2	PCR
Norrbom et al., 2011	8M	24.0 ± 1.8	Knee extension; 45 min; 26% of peak load.	Pressure chamber; 50 mmHg	PGC-1α	0&2	PCR

*M, Male; F, Female; RM, Repetition Maximum; PCR, Polymerase Chain Reaction; WB, Western Blot; VO_2max_, Maximal oxygen uptake; HR_max_, Maximum heart rate; PGC-1α, Peroxisome proliferator-activated receptorγcoactivator-1α; VEGF, ascular endothelial growth factor; VEGFR2, Vascular Endothelial Growth Factor Receptor 2; HIF-1α, Hypoxia inducible factor-1α; eNOS, Endothelial nitric oxide synthase.*

### Sensitivity Analysis

Three steps were performed for the sensitivity analysis: changing the analysis model, effect size selection, and exclusion of individual studies. VEGF and PGC-1α indices were not significantly affected after the sensitivity test and the outcomes were stable. In contrast, the HIF-1α was highly sensitive and characterized by unstable results.

### Quality Assessment

Based on the NIH scale, nine of the 10 studies included in this review (Ameln et al., 2005; Drummond et al., 2008; Norrbom et al., 2011; Larkin et al., 2012; Taylor et al., 2016; Christiansen et al., 2018; Ferguson et al., 2018; Preobrazenski et al., 2020; Barjaste et al., 2021) recorded an overall quality rating of “Good” and only one study (Norrbom et al., 2004) had an overall quality rating of “Fair” as shown in Table 2.

**TABLE 2:** Depiction of the risk of bias assessment.

NIH Tool	1	2	3	4	5	6	7	8	9	10	11	12	13	14	Total
Ameln et al., 2005	Y	N	Y	Y	N	N	N	NA*	N	N	Y	Y	Y	NA*	7/12
Barjaste et al., 2021	Y	Y	Y	Y	N	N	N	NA*	Y	N	Y	Y	Y	NA*	8/12
Christiansen et al., 2018	Y	N	Y	Y	N	N	N	NA*	Y	N	Y	Y	Y	NA*	7/12
Drummond et al., 2008	Y	Y	Y	Y	N	N	N	NA*	Y	N	Y	Y	Y	NA*	8/12
Ferguson et al., 2018	Y	N	Y	Y	N	N	N	NA*	Y	N	Y	Y	Y	NA*	7/12
Larkin et al., 2012	Y	Y	Y	Y	N	N	N	NA*	Y	N	Y	Y	Y	NA*	8/12
Norrbom et al., 2004	Y	N	Y	Y	N	N	N	NA*	N	N	Y	Y	Y	NA*	6/12
Norrbom et al., 2011	Y	Y	Y	Y	N	N	N	NA*	N	N	Y	Y	Y	NA*	7/12
Preobrazenski et al., 2020	Y	Y	Y	Y	N	N	N	NA*	N	N	Y	Y	Y	NA*	8/12
Taylor et al., 2016	Y	Y	Y	Y	N	N	N	NA*	Y	N	Y	Y	Y	NA*	8/12

Y = yes; N = no; NA = not applicable; * Not included in total score.

### Quantitative Synthesis

#### Vascular Endothelial Growth Factor and Vascular Endothelial Growth Factor Receptor-2

**FIGURE 2:**
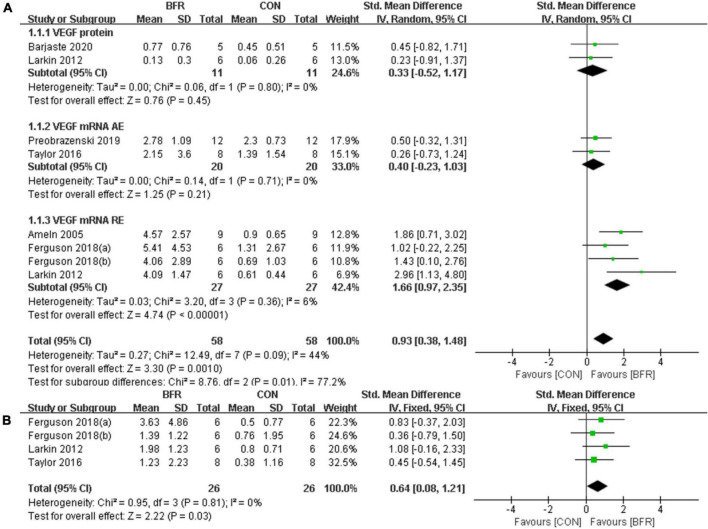
The forest plot of the effect size for studies assessing the effect of blood flow restriction exercise on the VEGF **(A)** and VEGFR-2 **(B)** mRNA.

Figure 2A shows the six studies (Ameln et al., 2005; Larkin et al., 2012; Taylor et al., 2016; Ferguson et al., 2018; Preobrazenski et al., 2020; Barjaste et al., 2021) that evaluated the effects of BFR on VEGF, whereas the three studies (Larkin et al., 2012; Taylor et al., 2016; Ferguson et al., 2018) reporting the effects of BFR on VEGFR-2 mRNA are presented in Figure 2B. The meta-analysis revealed that BFR group (*n* = 58) improved VEGF more significantly (*P* < 0.05) compared to the CON group (*n* = 58) (heterogeneity test, *P* = 0.09, I^2^ = 44%; SMD, 0.93 [0.38, 1.48]). VEGF protein (heterogeneity test, *P* = 0.80, I^2^ = 0%; SMD, 0.33 [−0.52, 1.17]), VEGF mRNA AE (heterogeneity test, *P* = 0.71, I^2^ = 0%; SMD, 0.40 [−0.23, 1.03]) or VEGF mRNA RE (heterogeneity test, *P* = 0.36, I^2^ = 6%; SMD, 1.66 [0.97, 2.35]) showed greater homogeneity in the sub-group analysis. Likewise, a significant difference (*P* < 0.05) was observed in the VEGFR-2 mRNA between the BFR (*n* = 26) and CON (*n* = 26) groups (heterogeneity test, *P* = 0.81, I^2^ = 0%; SMD, 0.64 [0.08, 1.21]).

#### Hypoxia Inducible Factor 1α

**FIGURE 3:**
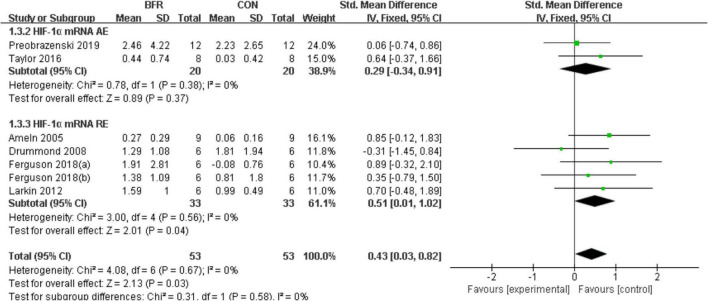
The forest plot of the effect size for studies assessing the effect of blood flow restriction exercise on the HIF-1α mRNA.

The effects of BFR on HIF-1α was investigated in six studies (Ameln et al., 2005; Drummond et al., 2008; Larkin et al., 2012; Taylor et al., 2016; Ferguson et al., 2018; Preobrazenski et al., 2020) as shown in Figure 3. HIF-1α was significantly improved (*P* < 0.05) in the BFR group compared to the CON group (*n* = 53) (heterogeneity test, *P* = 0.67, I^2^ = 0%; SMD, 0.43 [0.03, 0.82]). A greater homogeneity was detected in the sub-group analysis for HIF-1α mRNA AE (heterogeneity test, *P* = 0.38, I^2^ = 0%; SMD, 0.29 [−0.34, 0.91]) or HIF-1α mRNA RE (heterogeneity test, *P* = 0.56, I^2^ = 0%; SMD, 0.51 [0.01, 1.02]).

#### Peroxisome Proliferator-Activated Receptorγcoactivator-1α

**FIGURE 4:**
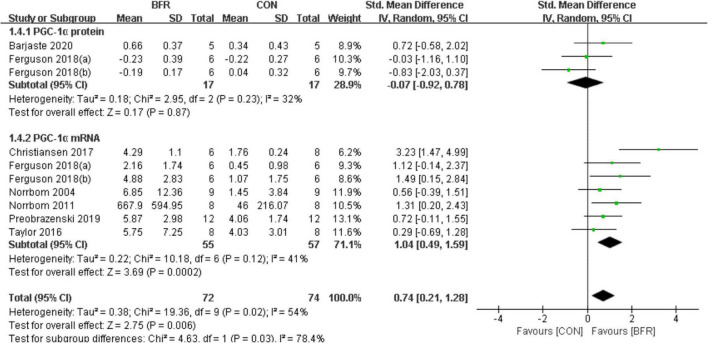
The forest plot of the effect size for studies assessing the effect of blood flow restriction exercise on the PGC-1α.

Seven studies (Norrbom et al., 2004, 2011; Taylor et al., 2016; Christiansen et al., 2018; Ferguson et al., 2018; Preobrazenski et al., 2020; Barjaste et al., 2021) reported the effects of BFR on PGC-1α (Figure 4). PGC-1α was significantly improved (*P* < 0.05) in the BFR group (*n* = 72) than the CON group (*n* = 74) (heterogeneity test, *P* = 0.02, I^2^ = 54%; SMD, 0.74 [0.21, 1.28]). Likewise, the sub-group analysis demonstrated a greater homogeneity for PGC-1α protein (heterogeneity test, *P* = 0.23, I^2^ = 32%; SMD, −0.07 [−0.92, 0.78]) or PGC-1α mRNA (heterogeneity test, *P* = 0.12, I^2^ = 41%; SMD, 1.04 [0.49, 1.59]).

#### Endothelial Nitric Oxide Synthase

**FIGURE 5:**
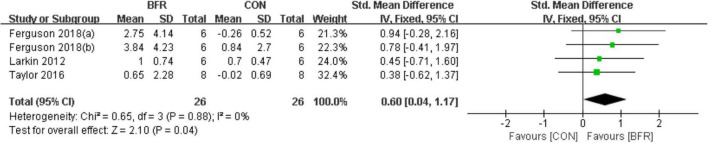
The forest plot of the effect size for studies assessing the effect of blood flow restriction exercise on the eNOS mRNA.

As shown in Figure 5, the effects of BFR on eNOS were evaluated in three of the reviewed articles (Larkin et al., 2012; Taylor et al., 2016; Ferguson et al., 2018). The meta-analysis revealed eNOS was more significantly improved (*P* < 0.05) in the BFR group (*n* = 26) compared to the CON group (*n* = 26) (heterogeneity test, *P* = 0.88, I^2^ = 0%; SMD, 0.60 [0.04, 1.17]).

### Analysis of Publication Bias

**FIGURE 6:**
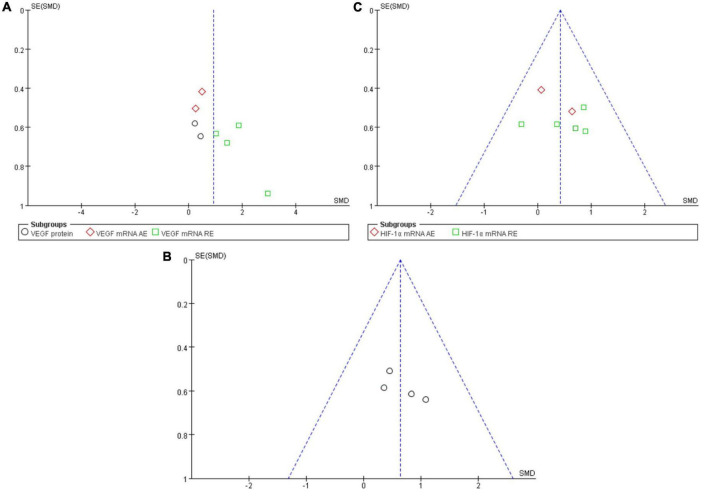
Funnel plot of publication bias for the effect of resistance exercise on the VEGF **(A)**, VEGFR-2 **(B)** and HIF-1α **(C)**.

A funnel plot was employed in analyzing the publication bias. Using the minimum requirement of the funnel plot, the total sample size of all the 10 studies reflected the publication bias to a certain degree. The feasibility of performing funnel plot analysis using a small sample size has been demonstrated by Lu et al. (2020) in a previous study. Figure 6 depicted the funnel plots of all the symmetrically-distributed indicators, reflecting a small degree of bias in the studies.

## Discussion

Studies in improving skeletal muscle capillary networks and functions emphasize the high intensity of either aerobic or resistance exercise (Prior et al., 2003; Gavin et al., 2007; Larkin et al., 2012). However, high-intensity exercise is restricted to some special populations, including cardiovascular patients and older persons. BFR exercise is a proposed alternative for traditional high-intensity exercise (Drummond et al., 2008) and it is important to compare their effects on skeletal muscle capillary growth. Hence, the present meta-analysis investigated the expression of VEGF, VEGFR-2, PGC-1α, HIF-1α, and eNOS in healthy adults after both exercise modalities. The results showed that the expression of the above mentioned angiogenic genes was significantly increased in the BFR group compared to the control group. However, different exercise programs may cause various changes in the mRNA expression of VEGF and HIF-1α. The present results revealed that resistance exercise can significantly increase the mRNA expression of HIF-1α and VEGF in healthy adults. Conversely, these positive effects were lacking for aerobic exercise.

It is speculated that this may be caused by different degrees of hypoxia, and the increase of HIF-1 may mainly depend on anaerobic metabolism (Lundby et al., 2009). In exercise with BFR, resistance exercise mainly stimulates the skeletal muscle system, which may lead to lower oxygen consumption than aerobic exercise (Ferrari et al., 2018). The lower oxygen exchange level may be the reason for the higher expression of HIF-1 when resistance exercise combined with BFR. Furthermore, some evidence suggests that resistance exercise with BFR may lead to better superposition effect on angiogenesis rather than aerobic exercise (Mueller et al., 2014; Conceicao et al., 2016; Mitchell et al., 2019). Mitchell et al. (2019) indicated that the change of capillary density after four weeks of aerobic training with BFR was similar to that without BFR. Briefly, resistance exercise combined with BFR may have a better effect on angiogenesis than aerobic exercise.

Interestingly, the included studies presented contradicting outcomes in the expression of VEGF (Larkin et al., 2012) and PGC-1α (Ferguson et al., 2018) protein from mRNA. The authors attributed the discrepancies to the limitations of sampling time as it might take several days after exercise for some proteins to be translated (Gustafsson et al., 2005; Perry et al., 2010). In contrast, Barjaste et al. (2021) reported that VEGF, HIF-1α, and PGC-1α protein expression were significantly increased at 3-h post-BFR-walking. This finding corroborates an animal-based study, which reported an increase in PGC-1α protein level in BFR-low intensity aerobic training (Bahreinipour et al., 2018). This might be explained by the different exercise modes adopted in the studies, reflecting diverse metabolic stress and mechanical tension between them. For instance, it was found the expression of HIF-1α, VEGF and PGC-1α proteins (Conceicao et al., 2016) was better stimulated by low-intensity BFR walking compared to low-intensity BFR cycling (Barjaste et al., 2021).

One of the central pro-angiogenic factors during exercise training is VEGF (Hellsten and Hoier, 2014) and its mRNA expression increases after a single bout of exercise (Gustafsson et al., 1999). This event was reported by Larkin et al. (2012) as the most notable transcriptional change among other angiogenic genes in response to the reduced blood flow and oxygen delivery in exercised skeletal muscle during BFR. The primary receptor of VEGF (VEGFR-2) mediates the VEGF-induced angiogenesis (Milkiewicz et al., 2005; Ferguson et al., 2018). Results from this meta-analysis indicated that VEGFR-2 mRNA increased after BFR-exercise, which may result from the increased VEGF binding (Ferguson et al., 2018) and the number of endothelial cells (Gustafsson et al., 2007) induced by BFR-exercise. Additionally, VEGF expression would be promoted by activating some of its key regulating factors (HIF-1α, PGC-1α, and eNOS) when BFR is combined with exercise, thereby eliciting an increased local muscular ischemia or hypoxia, shear-stress, and mechanical stress (Shweiki et al., 1992; Stein et al., 1995; St-Pierre et al., 2003; Bloor, 2005; Jager et al., 2007; Arany et al., 2008; Hawley et al., 2014; Paula et al., 2020).

HIF-1α is an important angiogenic regulator of hypoxia and metabolic stress (Semenza, 2006; Taylor et al., 2016). While skeletal muscle PO_2_ is reduced by exercise, exercise-dependent reduction in oxygen tension might facilitate up-regulation of HIF-1α expression in skeletal muscles. The levels of HIF-1α is increased significantly after BFR-exercise compared to non-BFR exercise (Larkin et al., 2012; Taylor et al., 2016; Barjaste et al., 2021). This reflects a higher degree of hypoxia, followed by increased activation of VEGF, which is the downstream factor of the HIF-1α pathway. This is in line with the present pooled data meta-analysis but contradicts the findings from the analysis of some included studies. Ameln et al. (2005) found no significant differences between BRF and non-BFR conditions in HIF-1α protein levels although the lactate levels indicated a lower oxygen tension during BFR-exercise. The degree of hypoxia may be already low enough to stimulate HIF-1α expression at 50 to 60% of the maximum work rate during non-BFR exercise. Hence, the intensity of exercise protocols may also play an important role in activating the HIF-1α by BFR-exercise. Other possible reasons that may likely account for the negative results include subject variability, exercise modes and sampling time (Drummond et al., 2008; Ferguson et al., 2018; Preobrazenski et al., 2020). Given these negative results, the enhanced angiogenesis induced by BFR more likely depends on the PGC-1α pathway.

PGC-1α has recently emerged as an inducer of angiogenesis in skeletal muscle, which strongly induces VEGF expression (Jung and Kim, 2014) in response to ischaemia (Arany et al., 2008). Therefore, increased oxidative stress (Kemp et al., 2003; Yu et al., 2008; Zhong et al., 2011) and fiber type-dependent AMPK signaling (Norrbom et al., 2011; Christiansen et al., 2018; Preobrazenski et al., 2020) may be associated with augmentation of BFR in exercise-induced PGC-1 mRNA. Four out of five studies including PGC-1α mRNA measurement showed a higher expression of the protein during BFR-exercise (Norrbom et al., 2011; Christiansen et al., 2018; Ferguson et al., 2018; Preobrazenski et al., 2020), which is consistent with the current pooled data meta-analysis. However, one study reported no differences between the two exercise modalities, which might be due to different exercise and BFR modes adopted (Taylor et al., 2016). Christiansen et al. (2018) found that muscle hypoxia was not a key factor for BFR upon comparing the effects of BFR and systemic hypoxia (∼3,250 m) on PGC-1α. Similarly, a previous study discovered that moderate-intensity cycling at simulated altitude (3,000 m) did not affect the PGC-1α mRNA in the skeletal muscle (Slivka et al., 2014). In addition, the alterations in the expression of PGC-1α protein might be influenced by the intensities of exercise with BFR (Bahreinipour et al., 2018; Ferguson et al., 2018; Barjaste et al., 2021). Moreover, this protein may also be affected by the sampling time because the increase in protein concentrations or levels may delay for hours or days after the exercise (Baar et al., 2002).

The binding of VEGF to VEGFR-2 activates a signaling cascade leading to NO production (Larkin et al., 2012). Furthermore, the production of NO is directly induced by the eNOS activity, which is controlled by either shear-stress dependent or independent VEGF expression (Larkin et al., 2012). Therefore, the increased eNOS mRNA expression after BFR-exercise (Ferguson et al., 2018) might result from the shear stimulus caused by BFR, as well as mechanical compression by skeletal muscle contraction. In contrast, Larkin et al. (2012) and Taylor et al. (2016) indicated that muscle expression of eNOS was not increased by the combination of BFR with acute low-intensity exercise. These inconsistent results may be due to variations in sampling time, as the peak expression of eNOS may appear before VEGF and VEGFR-2 (Milkiewicz et al., 2005; Ferguson et al., 2018).

### Study Limitations

Most of the studies included in this review reflected a small sample size and only 10 articles met the inclusion criteria. This reflects the lack of large sample size literature on this subject. Secondly, differences in muscle fiber types may explain the differences in the expression patterns of angiogenic factors, but there is a lack of direct evidence. Only two indirect evidences showed that there was no difference in the expression of AMPK (Christiansen et al., 2018) and PGC-1α (Norrbom et al., 2004) after exercise with blood flow restriction compared without blood flow restriction. Additionally, the changes of angiogenesis related factors in skeletal muscle can not really represent the actual angiogenesis. Capillary-to-fiber ratio is an effective index to reflect vascular density, however the relationship between blood flow restriction training intervention and capillary-to-fiber ratio is not clear. This limits our further explanation and verification of the effect of blood flow restriction training. In future research, we can further explore the effect of blood flow restriction training on vascular density and its relationship with angiogenesis related factors. Furthermore, the majority of studies focused on male samples while only three females were included. This might be attributed to the fact that women are more reluctant to accept muscle biopsy. Moreover, the forms of BFR used in various studies vary greatly, including cuff, raised limb and pressure chamber. This may lead to differences in the actual pressure applied and some errors may occur in horizontal comparison. Finally, most studies focused on mRNA expression and only a few considered protein alterations. Future studies should further explore the effect of BFR training on the expression of angiogenesis-related proteins, especially after a few days of exercise.

## Conclusion

This study revealed that exercise with BFR elicited more VEGF, VEGFR-2, HIF-1α, PGC-1α, and eNOS mRNA expression than exercise without BFR, but not VEGF and PGC-1α protein expression. Given that the combination of resistance exercise and BFR was more conducive to improving VEGF and HIF-1α mRNA expression compared to aerobic exercise. Therefore, BFR training may be more conducive to improve vascular function, the protocol should be considered when developing sports-based training programs. Results cannot be extrapolated to all individuals. Future studies should include samples from other populations to determine feasible training programs.

## Data Availability Statement

The original contributions presented in the study are included in the article/supplementary material, further inquiries can be directed to the corresponding author/s.

## Author Contributions

LW conceptualized and designed the study, collected and organized the data, and drafted the initial manuscript. ShuL collected and organized the data, reviewed the included articles, and conducted the analyses. ShiL and WY collected and organized the data and reviewed the included articles. WL and HQ conceptualized and designed the study and critically reviewed and revised the manuscript. TL conceptualized and designed the study, coordinated and supervised data collection, and critically reviewed and revised the manuscript. All authors read and approved the final manuscript.

## Conflict of Interest

The authors declare that the research was conducted in the absence of any commercial or financial relationships that could be construed as a potential conflict of interest. The handling editor declared a shared affiliation with several of the authors LW, HQ, TL, and WL at time of review.

## Publisher’s Note

All claims expressed in this article are solely those of the authors and do not necessarily represent those of their affiliated organizations, or those of the publisher, the editors and the reviewers. Any product that may be evaluated in this article, or claim that may be made by its manufacturer, is not guaranteed or endorsed by the publisher.

## Footnotes

1
https://www.nhlbi.nih.gov/health-topics/study-quality-assessment-tools

## Appendix

**APPENDIX A:** Search strategy.

Databases	Search strategy	Result (Approximately)
Scopus	#1: Title-Abs-Key (blood flow restriction or kaatsu or blood flow restricted) #2: Title-Abs-Key (exercise or training) #3: Title-Abs-Key (VEGF or HIF or PGC or NOS) #4: #1 and #2 and #3 Limiters - Published Date: 20010101-20210618	3,532 1,374,656 5,126,702 113
Pubmed	#1: [Title/Abstract] blood flow restriction or kaatsu or blood flow restricted #2: [Title/Abstract] exercise or training #3: [Title/Abstract] VEGF or HIF or PGC or NOS #4: #1 and #2 and #3 Filters: Publication date from 2001/01/01 to 2021/6/18	824 512,592 112,029 22
Web of Science	#1: TOPIC: (blood flow restriction or kaatsu or blood flow restricted) #2: TOPIC: (exercise or training) #3: TOPIC: (VEGF or HIF or PGC or NOS) #4: #1 and #2 and #3 Refined by: PUBLICATION YEARS: (20210618-20010101) Indexes=SCI-EXPANDED, SSCI, CCR-EXPANDED,	6,176 1,069,019 138,289 37
EBSCO	#1: Abstract: (blood flow restriction or kaatsu or blood flow restricted) #2: Abstract: (exercise or training) #3: Abstract: (VEGF or HIF or PGC or NOS) #4: #1 and #2 and #3 Year: 20010101-20210618	1136 2,983,417 221,179 30
